# Engineering of entanglement and spin state transfer via quantum chains of atomic spins at large separations

**DOI:** 10.1038/s41598-018-32145-3

**Published:** 2018-09-20

**Authors:** Dmitry I. Bazhanov, Ilia N. Sivkov, Valeri S. Stepanyuk

**Affiliations:** 1 Max Planck Institute of Microstructure Physics, Halle, 06120 Germany; 2 Faculty of Physics, Moscow State University, GSP-1, Lenin Hills, 119991 Moscow, Russia; 3 Institution of Russian Academy of Sciences Dorodnicyn Computing Centre, FRC CSC RAS, Vavilov st. 44, 119333 Moscow, Russia; 4 University of Zürich, Department of Chemistry, Winterthurerstrasse 190, CH8057 Zürich, Switzerland

## Abstract

Several recent experiments have shown that long-range exchange interactions can determine collective magnetic ground states of nanostructures in bulk and on surfaces. The ability to generate and control entanglement in a system with long-range interaction will be of great importance for future quantum technology. An important step forward to reach this goal is the creation of entangled states for spins of distant magnetic atoms. Herein, the generation of long-distance entanglement between remote spins at large separations in bulk and on surface is studied theoretically, based on a quantum spin Hamiltonian and time-dependent Schrödinger equation for experimentally realized conditions. We demonstrate that long-distance entanglement can be generated between remote spins by using an appropriate quantum spin chain (a quantum mediator), composed by sets of antiferromagnetically coupled spin dimers. Ground state properties and quantum spin dynamics of entangled atoms are studied. We demonstrate that one can increase or suppress entanglement by adding a single spin in the mediator. The obtained result is explained by monogamy property of entanglement distribution inside a quantum spin system. We present a novel approach for non-local sensing of remote magnetic adatoms via spin entanglement.

## Introduction

In diverse systems, long-range interactions between magnetic adatoms on surfaces were investigated both theoretically and in experiments^1–11^. Indirect coupling can be mediated by surface and bulk electrons(RKKY), superexchange or can be of dipole-dipole origin. For large distances between adatoms (>10−15 Å) the dipolar coupling is weak because it decreases as 1/*r*^3^. The rich physics caused by long-range interaction was discovered in many experiments^11^. For example, RKKY interaction between adatoms on metal surfaces can lead to collective ferromagnetic or antiferromagnetic ground states^12–14^. The 2*D* Kondo lattice was for the first time formed based on RKKY interaction between spins^15^. The presence of ferromagnetic coupling of Fe adatoms on Au(111) (the distance between Fe adatom is 1.3 *nm*) connected by organic molecules at large distances was revealed^16^. The superexchange mechanism mediated by the organic linkers was suggested to lead to the long-distance magnetic interactions. Organic molecules containing a magnetic atom are promising for the 2*D* magnetic superlattice. For example, formation of Fe superlattice in metal-organic quantum box network was achieved^17^. Superexchange mediated ferromagnetic coupling in 2*D* Ni molecular network was reported^18^.

Low-temperature scanning tunneling microscopy (STM) studies have allowed to resolve the long-distance adatom-adatom interactions on metal surfaces up to 80 Å^7,8^. At separations (>30−40 Å) the exchange interaction between magnetic adatoms is very small (a few *μ*eV)^10,19^. In such case, the first excitation energy of the total system (adatoms + a surface) becomes very small or even vanishes. It means that for antiferromagnetically coupled two adatoms, where in the ideal case entanglement exists, even very small thermal fluctuation would destroy entanglement (which is extremely fragile against thermal fluctuation). It would be of paramount importance to create entanglement in above mentioned nanostructures at large inter-atomic separation. The question is how to realize the entanglement between weakly coupled magnetic adatoms at large distances. Antiferromagnetic (AF) chains were proposed to use for quantum entanglement between remote qubits and for information transfer between them^20–23^. Very recent experiments of Choi *et al*.^24^ have demonstrated the possibility to tune entanglement and Kondo effect in FeMn chains on thin insulating layer of Cu_2_N on Cu(001). Antiferromagnetic spin chains up to ten atoms were constructed by placing Fe and Mn atoms on a Cu_2_N surface layer with a scanning tunneling microscope. Remarkably, it was found that the Kondo screening of the full chain depends on the inter-spin entanglement in the chain. This experimental finding revealed an entanglement at short and intermediate distances through AF chains. Importantly, that entanglement can survive at low temperatures in atomic-scale nanostructure on such substrate.

The first experimental evidence of long-distance entanglement in AF chains was recently presented^25^. It was revealed by Sahling *et al*. that unpaired two S = 1/2 spins separated by 220–250 Å entangle through a collection of spin singlets made up of antiferromagnetic dimerized spin-1/2 chains in a bulk material, the strontium-copper oxide.

The concept of long-distance (*LDE*) and modular (*ME*) entanglement^26–29^ offers a great opportunity to unveil how quantum information can be transferred at large distances on surfaces. The basic idea of this approach is to use a quantum mediator which provides non-direct interaction between unpaired spins. For the generation of entanglement between remotely separated spins one needs to suppress entanglement between spins and the system mediator, i.e. to weaken spins-mediator coupling. It is essential to find a mediator system best suited for the generation of long-distance entanglement The existence of dominant AF correlations in the mediator is the main mechanism of *LDE*. In this sense, one-dimensional open AF spin chains are excellent candidates for a generation of *LDE*. The modular entanglement arises in modular system constituted by a set of interacting moduli. Formally, it could allow to entangle spins at very large distances by increasing the number of moduli and to link distinct quantum registers in a future quantum computers. A most interesting feature of *ME* is its enhanced stability against thermal decoherence compared to the case of simple *LDE*^27^.

Inspired by these schemes we investigate long-distance entanglement for a realistic physical conditions. Firstly, we study 1*D* AF dimerized linear chains in bulk materials experimentally studied by Sahling *et al*. Secondly, we construct the mediator system from antiferromagnetic Fe dimers on a Cu_2_N/Cu(100) surface following experiments of Bryant *et al*.^30^ on a local control of magnetic anisotropy and exchange interactions in dimers. Such system can be considered as quantum playground where one can study entanglement in the ground state and upon the effect of magnetic pulses in atomic scale nanostructures with realistic parameters. In both cases we reveal a strong entanglement between remote non-interacting spins at large distances. We present results on ground state entanglement and also for entanglement propagation in non-equilibrium process initiated by localized excitation of spins by magnetic pulses. We show how entanglement can be tailored changing interactions between spins and by magnetic pulses. We reveal spin-sensing effect: non-local sensing of non-directly coupled spins via entanglement. We found that entanglement between remote spins strongly depends on the number of spins in modular structures showing a strong even-odd effect. A crucial role of *MAE* in *LDE* is demonstrated.

## Results and Discussion

### Generation of entanglement in the ground state between remote spins in a Sr_14_Cu_24_O_41_ bulk

**Figure 1:**
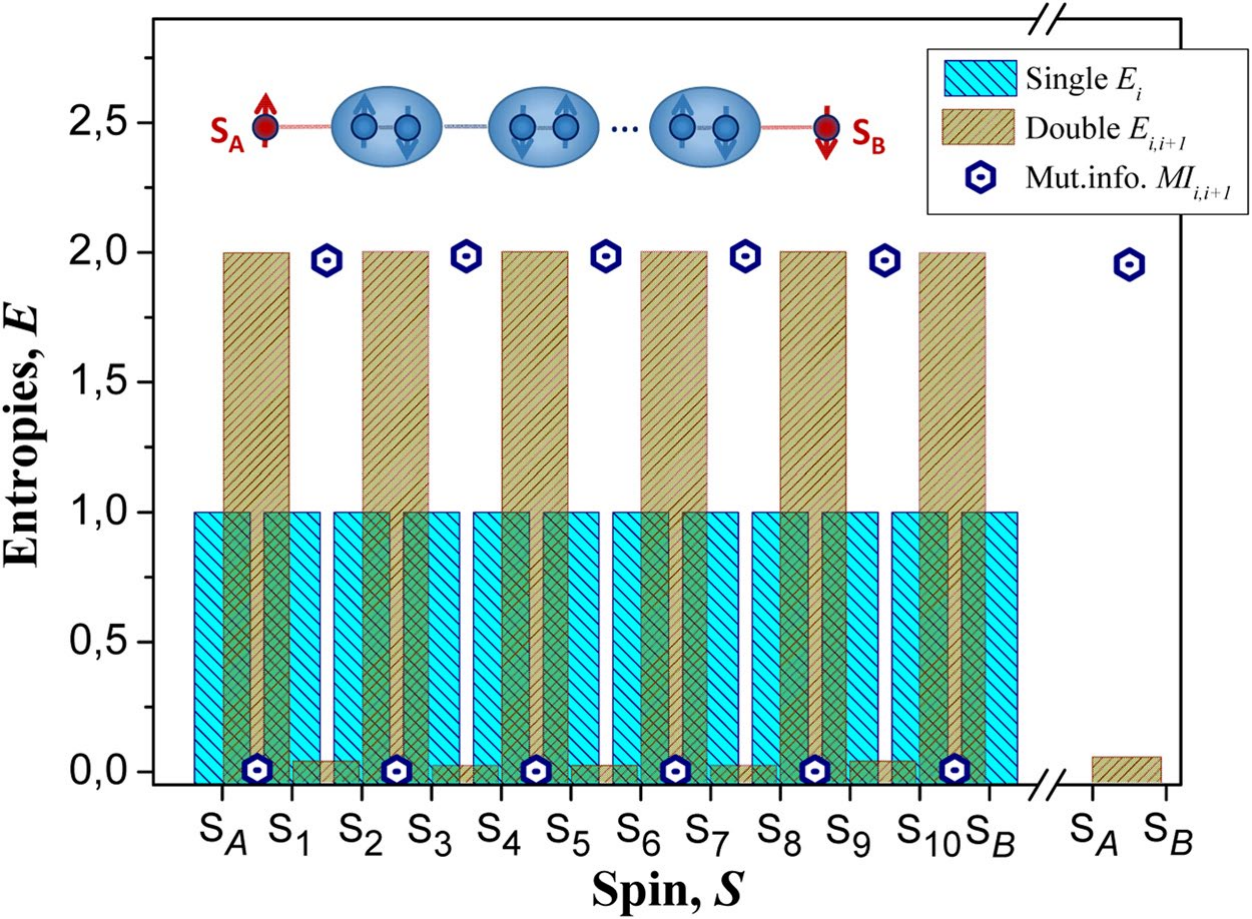
Inset: a schematic view of long-distance entanglement between *S*_*A*_ and *S*_*B*_ spins (in red) separated by antiferromagnetically coupled dimerized spin chain (in blue), as proposed in paper^25^. Distributions of single-site (*E*_*i*_), double-site (*E*_*i*,*i* + 1_) von Neumann entropies and double-site mutual information (*MI*_*i*,*i* + 1_) calculated for paired spins within a quantum spin chain made up of 12 spins.

**Figure 2:**
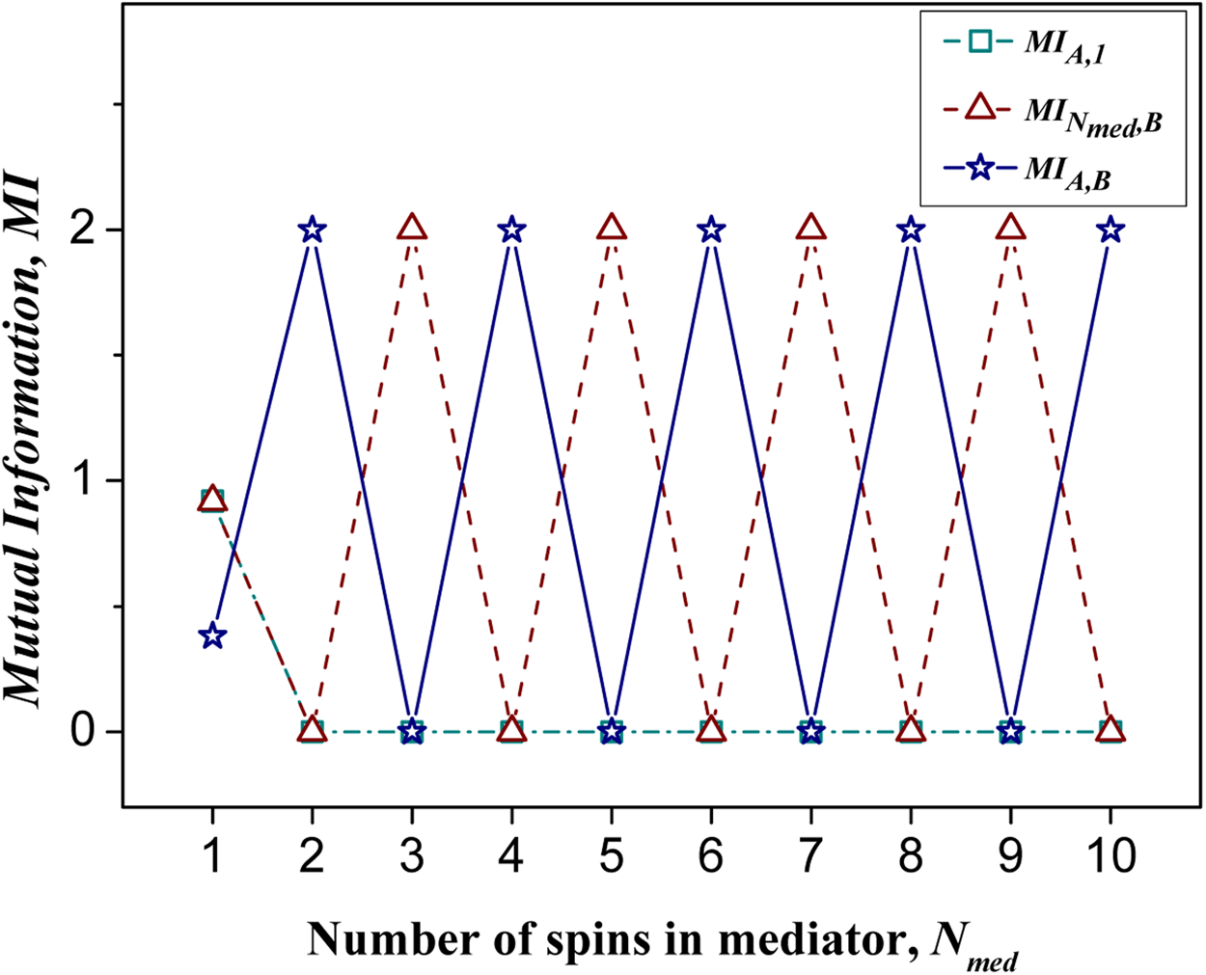
The double-site mutual informations *MI*_*A*,1_, $M{I}_{{N}_{med},B}$, *MI*_*A*,*B*_ calculated for spin pairs *S*_*A*_ − *S*_1_, *S*_*n*_ − *S*_*B*_ and *S*_*A*_ − *S*_*B*_, respectively, as a function of the number of spins, *N*_*med*_ (${N}_{med}=\overline{1,10}$), in a quantum mediator represented by dimerized spin chain.

Recent startling results published by Sahling and colleagues^25^ have shown a first evidence of experimental realization of *LDE* between remote noninteracting spins via antiferromagnetic quantum spin chains in a bulk material. Measuring both the magnetic susceptibility and specific heat capacity, as an entanglement witnesses, of a chain-like strontium-copper compound (Sr_14_Cu_24_O_41_) at low temperature and magnetic field, they revealed that unpaired spins of Cu atoms separated by several hundred angströms (~220–250 Å) exhibited quantum correlations (or quantum entanglement) via a chain of antiferromagnetically coupled spin-1/2 dimers below *T* ~ 2.1 *K*. The mechanism, predicted as the origin of these correlations between entangled spins, was ascribed by the inter-modulation potential between two various (spin-ladder and spin-chain) sublattice layers of Sr_14_Cu_24_O_41_. Besides, a simple model of 1*D* Hamiltonian based on Heisenberg interactions within antiferromagnetically coupled dimerized spin chain was applied to understand the obtained results. In the present paper, we will show numerically by means of QSH (Eq. 6), that a most relevant feature responsible for the generation of *LDE* between remotely separated spins (*S*_*A*_ and *S*_*B*_) and its propagation via a mediate quantum spin chain (the system quantum *mediator*), is the *monogamy* properties of quantum correlations within the spin system^29^. For the first time, the relation of monogamy with correlations was established by Coffman *et al*.^31^ for three-qubit pure states. It was shown that, unlike classical correlations, the quantum correlations cannot be strongly shared simultaneously between three qubits: if qubit *A* is fully entangled with qubit *B*, then qubit *A* cannot be simultaneously entangled with qubit *C*. Moreover, if *A* is not fully entangled with *B*, than *A* can have only a limited entanglement with *C*. Later on, a monogamy of entanglement was generalized by Osborne and Verstraete for pure and mixed states of an arbitrary number of qubits^32^. In setup, relevant to the case of quantum spin chain, it means: if spins within mediator are strongly entangled with each other, entanglement between spin on distant *A*(*B*) probe and the mediator will be weak for small exchange interaction between them, that should lead to a strong entanglement between *S*_*A*_ and *S*_*B*_ spins. The physics is the same in the case of antiferromagnetically coupled dimerized chain unveiled in strontium-copper oxide^25^: singlet states formed by dimers exhibit a strong entanglement and if the interaction between dimers is weak, *S*_*A*_ and *S*_*B*_ spins will be entangled, if they have a weak interactions with dimerized chain. To prove this statement, we present in Fig. 1 the distribution of mutual information (or entanglement) (Eq. 5) for neighboring spins within antiferromagnetically coupled dimerized Heisenberg spin-1/2 chain made up of *N*_*ch*_ = 12 spins, which were shared between *A*, *B* probes (*S*_*A*_ and *S*_*B*_) and the mediator (*S*_1_, …, *S*_10_), as a relevant example of the spin chain described in paper^25^. Based on that description, we applied for chain in our simulations the same sign and the coupling strength between spins in such a way, that there are two types of interactions: (1) a weak ferromagnetic interaction (*J*_1_ = 13*K*) between *A*(*B*) probe and the mediator and between dimers that compose the mediator; (2) a strong antiferromagnetic interaction (*J*_2_ = −115*K*) within these dimers. As shown in Fig. 1, the double-site MI, evaluated from plotted single and double-site von Neumann entropies (Eq. 5), demonstrates a large entanglement for antiferromagnetically coupled dimers (*S*_1_ − *S*_2_, *S*_3_ − *S*_4_, *S*_5_ − *S*_6_, *S*_7_ − *S*_8_, *S*_9_ − *S*_10_), but drops it drastically in between them for spin pairs (*S*_2_ − *S*_3_, *S*_4_ − *S*_5_, *S*_6_ − *S*_7_, *S*_8_ − *S*_9_) due to the weak interdimer interaction. Thereby, the mediator is represented by an entangled dimerized spin chain and its weak interaction with *A* and *B* probes, ascribed by small MI (*S*_*A*_ − *S*_1_, *S*_10_ − *S*_*B*_), leads to the strong quantum correlations (or *LDE*) of their spins (*S*_*A*_ − *S*_*B*_). We will demonstrate below, that much weaker interaction of *A* and *B* probes with the entangled mediator are capable to enhance *LDE* between these probes. Here, as shown in Fig. 1, the strength of generated *LDE* between probes is practically saturated. It is provided both by the ground state calculations of entanglement distribution and by the chosen ratio $|{J}_{1}/{J}_{2}| \sim 0.1$ of coupling strengths between spins in considered quantum spin chain. Owing to that, each antiferromagnetically coupled spin-1/2 dimers in the mediator approach their values of entropies and mutual information close to thermodynamic limit (*E*_*i*_ → 1, *E*_*i*,*j*_ → 0, *MI*_*i*,*j*_ → 2), corresponding to the maximally entangled pure singlet state. Thereby, two edge spins (*S*_*A*_ and *S*_*B*_) are forced to develop the strong quantum correlations towards the formation of a long-distance singlet state $|{{\rm{\Psi }}}^{AB}\rangle =1/\sqrt{2}(|\uparrow \downarrow \rangle -|\downarrow \uparrow \rangle )$ for entangled spins. However, *LDE* becomes extremely fragile against thermal fluctuation^29^. In order to avoid this situation, the compromise choice should be done: to increase the coupling strength between *A*(*B*) probe and the mediator and yet to hold it rather small to generate *LDE*. Besides that, previous theoretical studies have shown that *LDE* between distant probes depends on the size of the system mediator composed by sets of interacting spins or spin moduli^26,27^. Moreover, our recent work gave a clear evidence that the onset of entanglement and its propagation within antiferromagnetic spin chain can be tailored by the number (even or odd) of constituent spins (so-called parity effect)^33^. Based on that, it is worthy to know how *LDE* is described by monogamy of quantum correlations in presence of parity effect. In this sense, we considered here again a strongly correlated dimerized Heisenberg spin-1/2 chains up to ten spins as a quantum mediators in between two spin-1/2 probes, endowed by antiferromagnetically coupled spins with a same ratio of coupling strengths (${J}_{1} < 0,{J}_{2} < 0,|{J}_{1}/{J}_{2}| \sim 0.1$), as a good candidates to do this job. In Fig. 2 we plot an overall numerical results obtained for *MI*_*A*,*B*_ in the ground state as a function of the number of spins, *N*_*med*_, in a quantum mediator. It is well seen, that a large amount of entanglement between *A* and *B* probes (*MI*_*A*,*B*_ → 2) and, consequently, the onset of *LDE* between them are observed for the mediators with an even number of spins, while for their odd number counterparts the entanglement is vanished (*MI*_*A*,*B*_ → 0) and *LDE* is not created, except *N*_*med*_ = 1. By examining the entanglement distributions inside a spin chain for each *N*_*med*_, we found, remarkably, in odd-numbered mediators *N*_*med*_ ∈ {3;5;7;9} the vanishing correlations between adjacent *S*_*A*_ and *S*_1_ spins (*MI*_*A*,1_ → 0), whereas the correlations between an other pair of adjacent *S*_*n*_ and *S*_*B*_ spins are nonvanishing ($M{I}_{{N}_{med},B}\to 2$). It excludes the possibility to form *LDE* between *S*_*A*_ and *S*_*B*_ edge spins according to monogamy concept, as shown in Fig. 2.

### Generation of entanglement in the ground state between remote spins on a Cu_2_N surface

**Figure 3:**
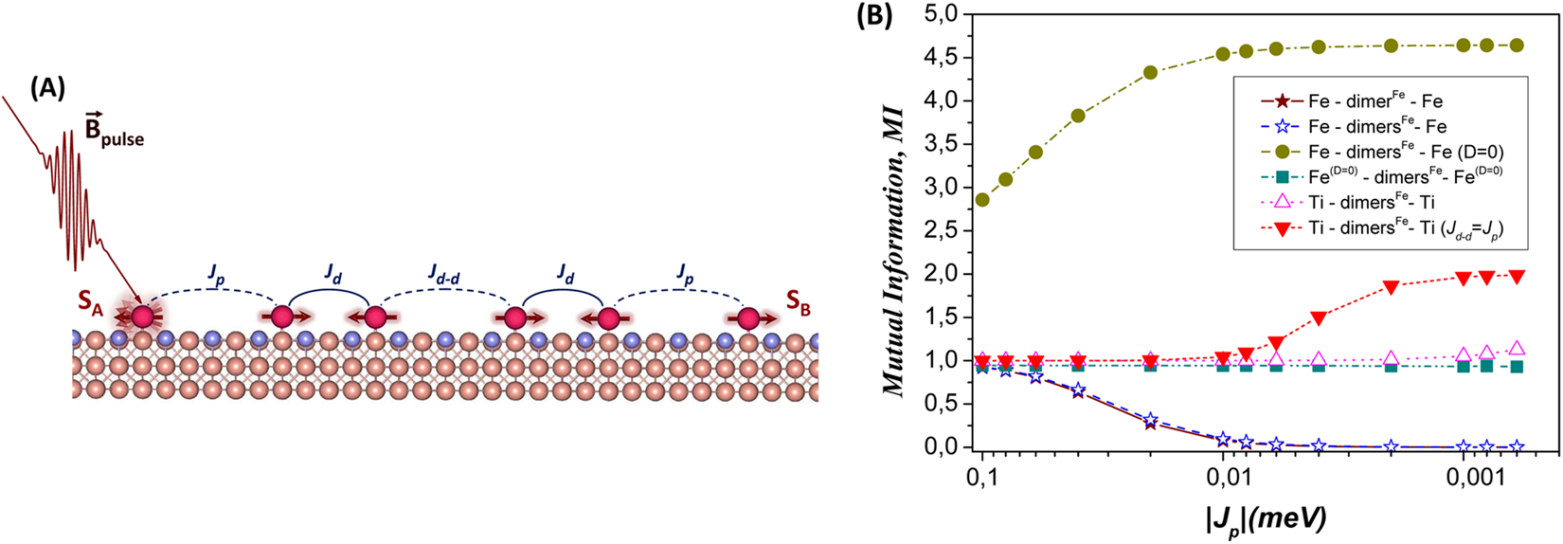
(**A**) Inset: A schematic illustration of the generation of long-distance entanglement between edge *S*_*A*_ and *S*_*B*_ spins of antiferromagnetic dimerized spin chain made up of 6 spins placed on a Cu_2_N/Cu(001) surface. The red (dark) spheres indicate individual atoms of spin chain, the blue (dark gray) spheres indicate the N atoms and the orange (light gray) ones indicate Cu atoms, respectively. The exchange interactions within a spin chain indicate the spin couplings between *S*_*A*_(*S*_*B*_) spin and dimer (*J*_*p*_), within dimer (*J*_*d*_) and between dimers (*J*_*d* − *d*_), respectively. The time-dependent external magnetic pulse (${\overrightarrow{B}}_{pulse}$) applied to *S*_*A*_ spin in order to trigger the spin dynamics in considered chain; (**B**) Double-site mutual information (*MI*) between *A* and *B* probes (*A* ≡ *B* ∈ {*Fe*,*Ti*}) as a function of spin coupling strength (*J*_*p*_) between these probes and a quantum mediator represented by one (*dimer*^*Fe*^) or two (*dimers*^*Fe*^) Fe dimers within a quantum spin chain on a Cu_2_N/Cu(001) surface. ‘(D = 0)’ means no magnetic anisotropy of individual atomic spins or the whole spin chain is involved in numerical calculations of *MI*.

**Figure 4:**
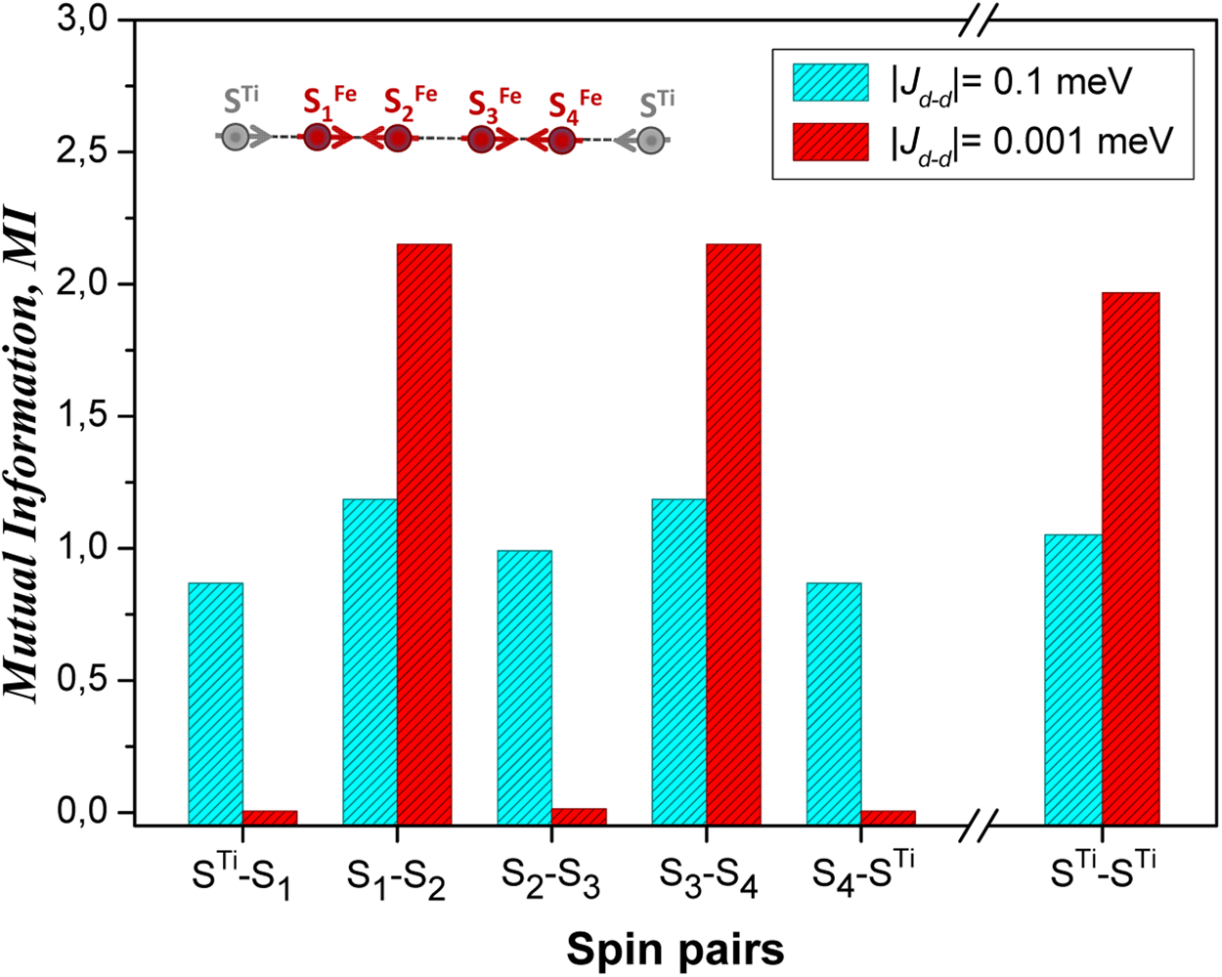
Distribution of double-site mutual information (*MI*) for paired spins within a quantum spin chain made up of 6 spins calculated for interdimer interactions |*J*_*d* − *d*_| = 0.1 *meV* and |*J*_*d* − *d*_| = 0.001 *meV*, respectively, at fixed interaction |*J*_*p*_| = 0.001 *meV*. Inset: a schematic view of quantum spin chain.

Now, we propose a scheme how to realize the entanglement between remote magnetic probes at large separations on surfaces. We demonstrate here that *LDE* between varied noninteracting *S*_*A*_ and *S*_*B*_ spins can be generated using an appropriate quantum mediator, composed by sets of antiferromagnetically coupled spin dimers under experimentally realistic conditions. Recent remarkable experiments of Choi *et al*.^24^ on Kondo systems revealed the onset of entanglement in AF compact FeMn chains on a thin insulating layer of Cu_2_N/Cu(001) surface even in the presence of decoherence caused by the local immediate environment. It was shown, that these spin chains with defined entanglement can be produced by changing their composition and coupling strength in a control way via atomic manipulation with STM. Besides, recent engineering^30,34^ of Fe and Co dimers on a Cu_2_N surface layer have demonstrated the ability to tune locally with STM the sign and strength of their exchange interactions and magnetic anisotropies by adjusting the relative positions of their constituent atoms on the surface. These experimental findings give rise an opportunity to employ such spin coupled magnetic dimers as a perspective building units (or spin moduli) for simulating a quantum mediator in between remote magnetic probes *A* and *B* on a Cu_2_N/Cu(001) surface. Here, we focus only on the most elementary mediators composed by one and two Fe dimers, respectively. Each dimer contains two antiferromagnetically coupled spins in a quantum state *S*^*Fe*^ = 2 and far apart separated (~7.2 Å) by superexchange interaction in [100] direction on a Cu_2_N surface layer^30^. Having built such mediators, we employ them as representative parts of supported quantum spin chains made up of *N*_*ch*_ = 4 and *N*_*ch*_ = 6 spins, respectively, in order to examine the onset of entanglement in the ground state between *A* and *B* probes far-separated on surface. For this purpose, three types of interactions are considered within these chains: (1) a weak variable antiferromagnetic interaction (−0.1 *meV* < *J*_*p*_ < 0) between *A*(*B*) probe and the mediator; (2) a relatively strong antiferromagnetic interaction (*J*_*d*_ = −0.7 *meV*) within each Fe dimer of the mediator, endowed by the measured values of magnetic anisotropy (*D* = −1.87 *meV* and *E* = 0.31 *meV*)^30^; (3) a weak antiferromagnetic interdimer interaction (*J*_*d* − *d*_ = −0.1 *meV*) within the mediator (see sketch in Fig. 3A). It is worthy to note here, that the sign and coupling strength of *J*_*p*_ and *J*_*d* − *d*_ interactions are chosen in such a way as to provide both the relevant experimental findings of long distance interactions ($|J|\sim 1.0\,meV\,\Longleftrightarrow \,d\sim 10\,\AA$) between individual atomic spins on a Cu_2_N^34,35^ and monogamy (or small enough ratio $|{J}_{p}/{J}_{d}|\lesssim 0.1$) of their quantum correlations leading to *LDE*^29^. Based on that choice of interactions, the magnetic probes *A* and *B* can be sufficiently far apart from each other (*d*_*AB*_ > 27 Å) on a Cu_2_N/Cu(001) surface. First of all, we treat in *A* and *B* locations the spins ascribed by single Fe atoms on a Cu_2_N^36^. In this case, the probes *A* ≡ *B* ∈ {*Fe*} and their spins are in the same quantum state *S*_*A*_ = *S*_*B*_ = *S*^*Fe*^ = 2 with magnetic anisotropy (*D* = −1.55 *meV* and *E* = 0.31 *meV*) similar to that for spins in a quantum mediator. Thus, the considered quantum spin chains are represented, so far, by strongly correlated antiferromagnetically coupled dimerized Heisenberg spin-2 chains, which, we expect, can exhibit *LDE* in the ground state between far-separated *S*_*A*_ and *S*_*B*_ edge spins on a Cu_2_N. The onset of *LDE* for both spin chains, expressed by double-site mutual information (*MI*_*Fe*,*Fe*_ ≡ *MI*_*A*,*B*_), is reported in Fig. 3B as a function of coupling strength *J*_*p*_ between Fe probes and the mediator. The obtained data clearly show that *LDE* values actually coincide for both chains. This result confirms, remarkably, the statement reported before that the same ground-state entanglement arises inside the spin systems composed by a set of identically interacting spin moduli of a fixed size^27^. Therefore, we will present further in Fig. 3B the ground-state numerical calculations only for quantum spin chain made up of *N*_*ch*_ = 6 spins, as a general case. As it is shown, *LDE* is decreasing rapidly and monotonically with *J*_*p*_ decay, starting from its maximum value ($M{I}_{Fe,Fe} \sim 1$) and vanishing asymptotically in the limit of weak coupling (*MI*_*Fe*,*Fe*_ → 0). We interpret such behavior of spin entanglement by means of recent experimental finding, reported for strongly correlated AF chains on a Cu_2_N^24^, which showed that the ratio of the coupling strength with magnetic anisotropy (*J*/|*D*|) can determine the degree of entanglement of spins. To confirm the impact of magnetic anisotropy, we have carried out, so-called, “Gedanken experiment” by “switching off” the magnetic anisotropies of individual iron spins inside considered spin chain. As a result, the successful generation of *LDE* between far-separated Fe probes is obtained now (see Fig. 3B). For comparison, *LDE* in this case is increasing monotonically with *J*_*p*_ decay and converging rapidly in the limit of weak coupling to the asymptotic value, corresponding to the maximally entangled spin-2 chain^37^. Thus, the obtained results indicate: if spins have magnetic anisotropy (*D*) the generation of entanglement is more complicated. For vanishing magnetic anisotropy the spin system is maximally entangled, while any anisotropy reduces the spin entanglement. It is worthy to note here, that the presence of transverse part of magnetic anisotropy (*E*) can vice versa promote the generation of spin entanglement, due to the quantum tunneling phenomenon^38^. As a result of the impact of magnetic anisotropy: decreasing the interaction between *A*(*B*) probe and the mediator does not guarantee increasing the entanglement between *A* and *B* probes. In this sense, it becomes apparent that the necessary condition to generate *LDE* between far-separated edge spins of a quantum spin chain is the trade off between coupling strength, governed by monogamy and magnetic anisotropy of constituent spins in a certain set of quantum states. To prove this statement, we continue “Gedanken experiment” by excluding the contributions from magnetic anisotropy of Fe probes only and examine the onset of *LDE* between them in such specified quantum spin chain. Figure 3B reports that, preserving the magnetic anisotropy in the chain mediator significantly suppressed the entanglement between Fe probes in comparison with their counterparts in spin chain without magnetic anisotropy. However, the probes still have a considerable degree of entanglement ($M{I}_{Fe,Fe} \sim 1$), which is conserved practically at the whole range of coupling strength *J*_*p*_. This result undoubtedly sustains the above statement and shows clearly how to realize a physically feasible *LDE* on a Cu_2_N/Cu(001) surface, namely the replacement of Fe probes by ones which exhibit at least a very small magnetic anisotropy. Spin-1/2 probes in this regard are ones of the best and particular desirable candidates, since they manifest readily a vanishing magnetic anisotropy in quantum spin systems. Among a versatile spin-1/2 probes we propose to use herein individual Ti atoms modeled as an *S*^*Ti*^ = 1/2 spins on a Cu_2_N, as determined by STM measurements^39^. Having done this, we built in this case a new spin chain with *A* ≡ *B* ∈ {*Ti*} probes, endowed by spins *S*_*A*_ = *S*_*B*_ = *S*^*Ti*^ = 1/2 antiferromagentically coupled via a quantum mediator composed by two Fe dimers. Note here, that recent theoretical study of R. Pushpa *et al*.^40^ reported about the ferromagnetic coupling of individual Ti spins mediated by superexchange interaction in [100] direction on a Cu_2_N/Cu(001) surface. Based on that, we performed the ground state calculations of *LDE* between Ti probes assuming their weak ferromagnetic interaction (0 < *J*_*p*_ < 0.1 *meV*) with a system mediator and found no much difference with *LDE* repeated in the antiferromagnetic case. Therefore, we constrained ourselves here for treatment of only ferromagnetic interaction without limiting the generality of obtained results and conclusions. The ground-state numerical calculations of double-site mutual information (*MI*_*Ti*,*Ti*_ ≡ *MI*_*A*,*B*_) for such quantum spin chain clear demonstrate the onset of *LDE* between far-separated Ti probes (see in Fig. 3B). A saturated *LDE* is observed with a large enough value ($M{I}_{Ti,Ti} \sim 1$), which increases slightly in the limit of weak coupling^27^. Further enhancement of *LDE* may be achieved for considered spin chain in terms of so-called ME, as introduced to the modular spin system^27^. For this purpose, we will increase dimerization of chain by changing its interdimer interaction in such a way that |*J*_*d* − *d*_| = |*J*_*p*_|. Remarkably for such modular spin chain, the rise of *LDE* is observed following the gradual convergence in the limit of weak coupling to the asymptotic value (*MI*_*Ti*,*Ti*_ → 2), which is almost twice as large as in the previous case of *LDE* (see Fig. 3B). To gain insight into that rise of *LDE*, we present in Fig. 4 the entanglement distribution between spin pairs within both modular spin chains with interdimer interactions *J*_*d* − *d*_ = −0.1 *meV* and *J*_*d* − *d*_ = −0.001 *meV*, respectively and look again at monogamy of their quantum spin correlations. We find that much weaker interdimer interaction in the modular spin chain increases significantly quantum correlations inside each spin dimer (*S*_1_ − *S*_2_, *S*_3_ − *S*_4_) of the chain mediator, that drops the correlations between the mediator and Ti probes, ascribed by negligible MI between them (*S*^*Ti*^ − *S*_1_, *S*_4_ − *S*^*Ti*^). Thereby, it leads consequently to the strongest quantum correlations (or *ME*) between edge Ti spins (*S*^*Ti*^ − *S*^*Ti*^) in terms of monogamy, as shown in Fig. 3B. Here, it is worthy to note, that far-separated Ti probes in modular spin chain reveal in the limit of weak coupling the maximally entangled pure singlet ground state |Ψ^*TiTi*^〉, as above-mentioned |Ψ^*AB*^〉 for antiferromagnetic spin-1/2 dimer, despite the presence of magnetic anisotropy in chain mediator. This result unveils a quiet promising way to design spin entanglement and information transfer via quantum spin chains on insulating supports.

### Non-equilibrium spin dynamics: spin sensing of remote spins on a Cu_2_N surface

**Figure 5:**
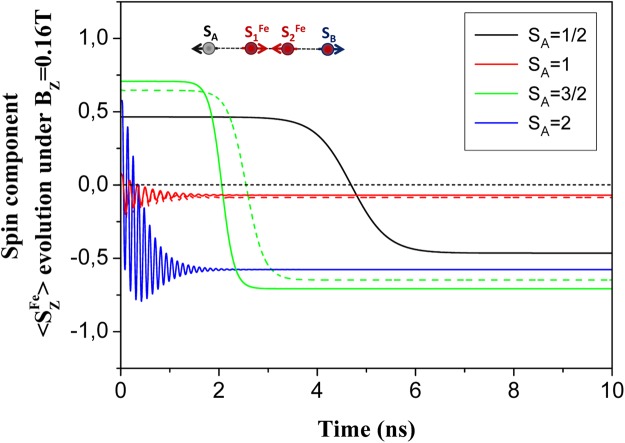
Time evolution of spin expectation $\langle {S}_{Z}^{Fe}\rangle$ of probe *B* ∈ {*Fe*} in dependence on the *S*_*A*_ spin value of probe *A* in 4-atomic quantum spin chain with exchange interactions *J*_*p*_ = −0.01 *meV* and *J*_*d*_ = −0.7 *meV* and under applied magnetic field *B*_1,*z*_ = 0.16*T*. Dotted lines represent spin dynamics corresponds to *S*_*A*_ = 1 and *S*_*A*_ = 3/2 spins, endowed by the magnetic anisotropy parameters (*D* and *E*) recalculated from available experimental data^62^, using rotation matrices^63^, within the coordinate axes (*x*,*y*,*z*) directions that we have followed in QSH (Eq. 6) for Fe spins in the chain mediator. Inset: a schematic view of a quantum spin chain.

**Figure 6:**
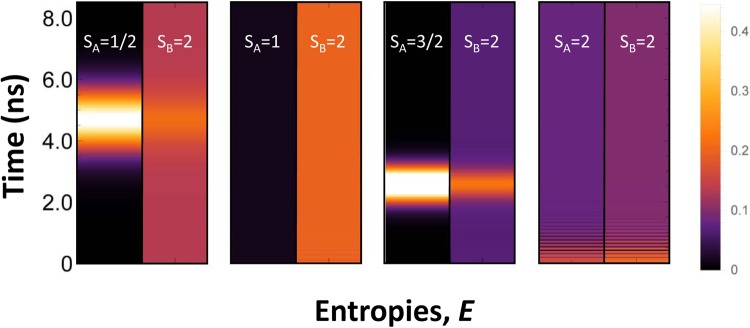
The time evolution of single-site Von Neumann entropies (*E*_*i*_) of probe spins *S*_*A*_ and *S*_*B*_ in 4-atomic quantum spin chain under magnetic field *B*_1,*z*_ = 0.16*T* applied to the spin *S*_*A*_ ≡ *S* ∈ {1/2; 1; 3/2; 2}, whereas the spin *S*_*B*_ = 2.

**Figure 7:**
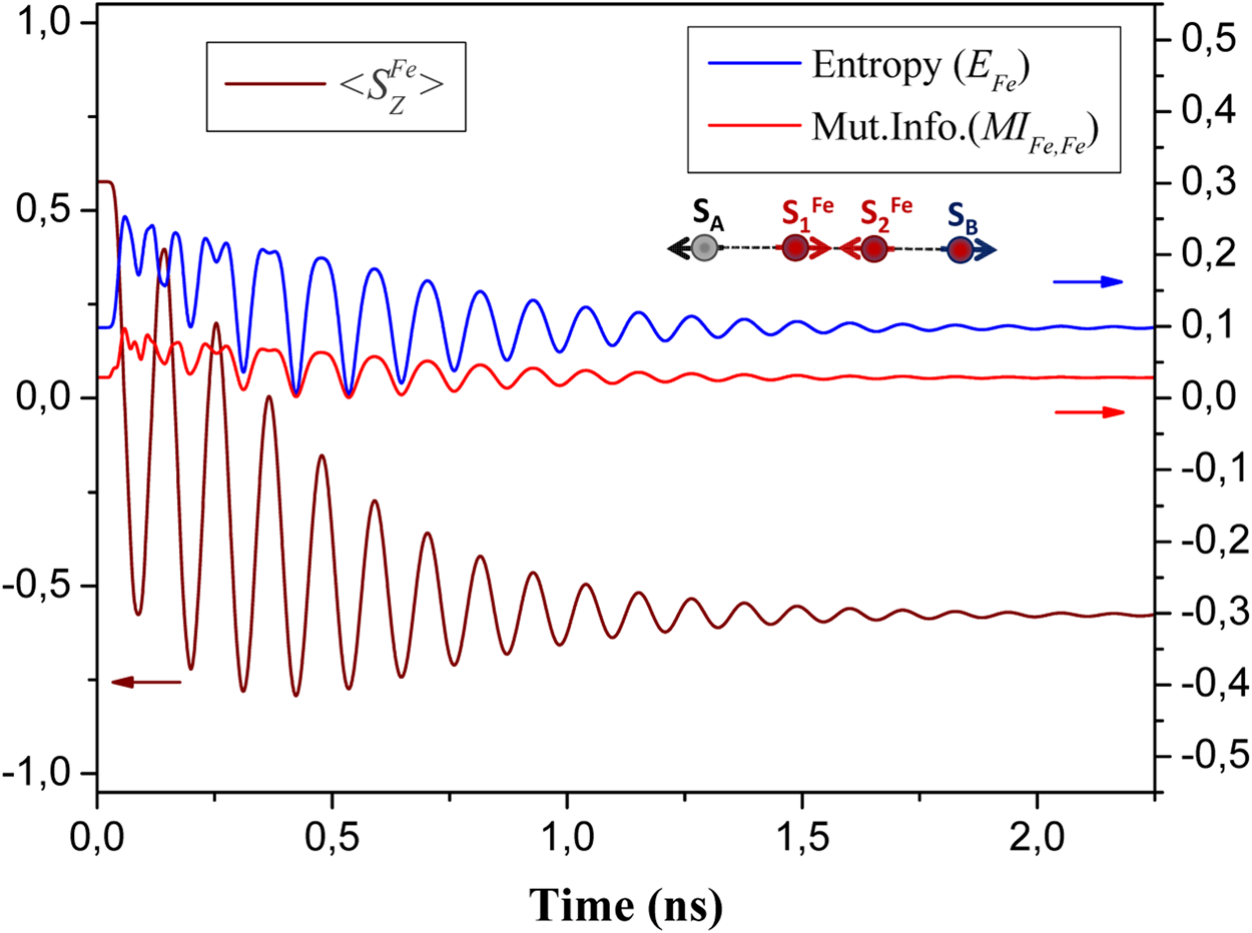
The time evolution of spin expectation $\langle {S}_{Z}^{Fe}\rangle$ and single-site entropy *E*_*Fe*_ on probe *B* and mutual information *MI*_*Fe*,*Fe*_ between *A* and *B* probes, where *A* ≡ *B* ∈ {*Fe*}. Inset: a schematic view of a quantum spin chain.

**Figure 8:**
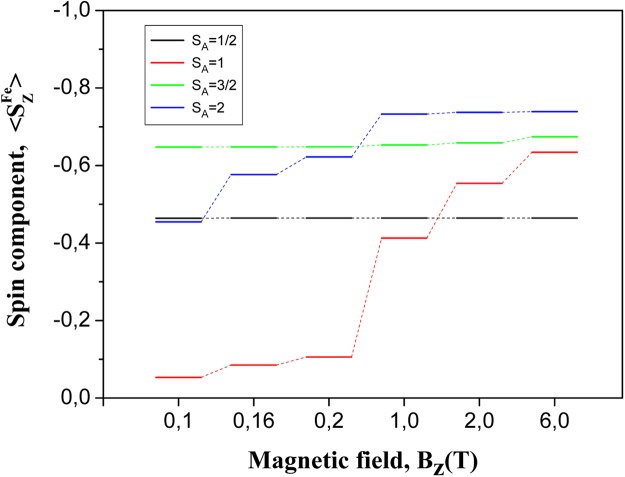
The dependence of the spin expectation $\langle {S}_{Z}^{Fe}\rangle$ values of probe *B* ∈ {*Fe*} on the external magnetic field *B*_*z*_ applied to probe *A* with the spin values *S*_*A*_ ∈ {1/2; 1; 3/2; 2} in a quantum spin chain made up of *N*_*ch*_ = 4 spins.

Now, we turn to the discussion of sensing for spins remotely separated on a surface. This problem has recently attracted a great attention. For example, recent experiments of Yan *et al*.^41^ reported on nonlocal magnetic sensing on a surface. They have demonstrated that only a few-atom spin systems can be used to sense magnetic states of single and multiple nano-antiferromagnets. Individual Fe atoms have been used as an electron spin resonance (*ESR*) sensor for remote sensing of spins in experiments of Choi *et al*.^42^. Here, we propose a novel approach for sensing of remote spins based on *LDE*. We have performed spin dynamics calculations for two probe spins *S*_*A*_ and *S*_*B*_ (see Fig. 5) supported on a Cu_2_N/Cu(001) surface and remotely separated via AF coupling with a quantum mediator composed by single Fe dimer, as described in previous section. We constrained ourselves here for treatment of only single Fe dimer in the mediator, since the mediator with couple spin dimers exhibit the same *LDE* between probe spins, whereas the time consuming for numerical calculations increases essentially. For simulation of spin sensing we kept *S*_*B*_ spin fixed to the state with quantum number *S*^*Fe*^ = 2, whereas *S*_*A*_ was changed in the range of the spin quantum numbers *S* ∈ {1/2; 1; 3/2; 2}. Results presented in Fig. 5 reveal that, the expectation value of *S*_*B*_ spin ($\langle {S}_{Z}^{Fe}\rangle$) depends on the *S*_*A*_ spin for both dynamical and static regimes, despite the large separation between them. One can see that, the time evolution of $\langle {S}_{Z}^{Fe}\rangle$ is significantly different for different spins at *A* location. In an other words, one can treat *S*_*B*_ spin as the spin sensor, which exhibits a non-local time-dependent sensing of the remote *S*_*A*_ spin. To give clear evidence that spin sensing is caused by entanglement in our system we present in Fig. 6 the time evolution of single-site Von Neumann entropies after the application of the pulse magnetic field of intensity *B*_1,*z*_ = 0.16*T* on *S*_*A*_ spin. These results unambiguously demonstrate that the single-site entropies and their time evolution strongly depend on quantum state of *S*_*A*_ spin, making sensing of remote spins possible. To gain more insight into the interplay between entanglement and the spin dynamics, we present in Fig. 7 the time evolutions of $\langle {S}_{Z}^{Fe}\rangle$ and single-site entropy *E*_*Fe*_ on probe *B* and mutual information *MI*_*Fe*,*Fe*_ between *A* and *B* probes (*A* ≡ *B* ∈ {*Fe*}). One can see that all of them exhibit an oscillatory behavior and maximum (minimum) of entropy and mutual information perfectly correspond to minimum(maximum) of the expectation value $\langle {S}_{Z}^{Fe}\rangle$. Note, that in the ground state (*B*_*Z*_ = 0) the expectation value $\langle {S}_{Z}^{Fe}\rangle$ = 0, if *S*_*A*_ is an integer spin due to entanglement of AF spins in chains^43^. For a half-integer *S*_*A*_ the spin entanglement is strongly reduced due to a suppression of quantum tunneling^38^. The expectation value $\langle {S}_{Z}^{Fe}\rangle$ can be tuned by changing the magnetic field acting on *S*_*A*_ spin, as well seen in Fig. 8. However, there is significant difference between spin dynamics and the response to the magnetic field for integer and half-integer spins on probe *A*. For an integer spins *S*_*A*_ ∈ {1; 2} the expectation value $\langle {S}_{Z}^{Fe}\rangle$ increases with increasing magnetic field *B*_*z*_ (approaching to a classical value of $|\langle {S}_{Z}^{Fe}\rangle |$ = 2), because the magnetic field reduces the spin entanglement. At the same time, the numerical calculations clearly show that for a half-integer spin *S*_*A*_ ∈ {1/2;3/2} a scenario of spin sensing in magnetic field *B*_*z*_ is different. Since for a half-integer spin the quantum tunneling is suppressed, it leads to a strong decreasing of the single site entropy on *S*_*A*_ spin even in small magnetic fields. However, the single-site entropies on all spins of the mediator are very sensitive to the spin on probe *A* (both for *S*_*A*_ = 1/2 and *S*_*A*_ = 3/2 spins). For example, under applied magnetic field *B*_1,*z*_ = 0.10*T* a single-site entropy of the first spin of the mediator more than two times larger for *S*_*A*_ = 1/2 than for *S*_*A*_ = 3/2. Thereby, in the case of the half-integer spins, spin sensing occurs due to spin correlations of *A* and *B* probes with a quantum mediator, where *S*_*B*_ spin has a strong entanglement with the edge spin of the mediator. This spin entanglement between *B* probe and the mediator is practically unchanged for all applied magnetic fields. Therefore, magnetic field applied to a half-integer *S*_*A*_ spin does not affect the spin expectation value $\langle {S}_{Z}^{Fe}\rangle$ in Fig. 8. The above results suggest that sensing of remote spins on surfaces could be possible using atomic-scale nanostructures, promoting *LDE*. With respect to possible experimental confirmation of our theoretical predictions we believe that spin-polarized STM, Kondo effect and *ESR* could be used to detect different spin states on distant spins by engineering an appropriate mediators for *LDE*.

Finally, we would like to comment on the influence of environment-induced decoherence on entanglement (spin decoherence of magnetic units on surfaces was recently reviewed by Delgado and Fernandez-Rossier^44^). As we have already noted, a thin insulating layer of Cu_2_N on Cu(001) significantly reduces decoherence in atomic-scale nanostructures and spin entanglement was detected at low temperatures^24,45^. Very long lifetime of spin states for antiferromagnetic chains on a Cu_2_N was found in experiments and explained by entanglement of spins in chains^46,47^. Additionally, in antiferromagnetically coupled quantum spin chains, studied in the present work, the stability of spin entanglement against thermal noise can be significantly enhanced by engineering of single-modulus parameters^22^. We suggest that future progress in preserving spin coherence on surfaces could be done using a technique known as dynamical decoupling^48–50^. Using optimal pulse sequence one can suppress decoherence. For example, dynamical decoupling, which uses stroboscopic spin flips, gives an effective zero coupling to the environment^50^. This approach has already been applied to several solid state systems, for example, diamonds with nitrogen vacancy centers^51^, phosphorous donors in a silicon system^52^ and proposed for molecular magnets^53^. We think that it deserves further applications for quantum spin chains supported on surfaces.

To conclude, experimental results on long-distance entanglement in spin chains in bulk indicated that antiferromagnetic dimers could serve as the system quantum mediator responsible for quantum information transfer between probe spins at large distances. We have performed ground-state numerical calculations for prototype quantum spin chain based on quantum spin Hamiltonian for the setup used in the experiment and revealed a strong entanglement between remote non-interacting edge spins. We have shown that increasing the number of constituent spins in the mediator can increase or suppress entanglement between edge spins. This result is explained by monogamy property of entanglement distribution inside a quantum spin chain. We have proposed to use antiferromagnetic Fe dimers on insulating layer of a Cu_2_N/Cu(001) surface as a perspective spin moduli for simulating a quantum mediator, which can provide a physical realization of long-distance entanglement between distant magnetic adatoms on surface. Further, we have demonstrated the possibility of non-local spin sensing at large spin-spin separations on surface. A strong effect of a magnetic field on sensing on integer spins is shown. Our studies pave the way for new experimental studies on entanglement among adatoms and atomic-scale nanostrucres on surfaces.

## Methods

To study the quantum spin dynamics of the system, we used the method proposed by Wieser^54^, where the time-dependent Schrödinger equation is solved together with the damping term as an analogue of the Landau-Lifshitz (LL) equation^55^ for classical magnetic dynamics. According to this approach the time-dependent Schrödinger equation with the relaxation term can be written as

1 $$i\hslash \frac{d}{dt}|\psi (t)\rangle =(\hat{H}-i\lambda (\hat{H}-{\langle H\rangle }_{t}))|\psi (t)\rangle ,$$

where *Ĥ* is a quantum spin Hamiltonian (QSH) and 〈*H*〉_*t*_ is an energy of the system at time *t*. It was shown^54^, that for the Heisenberg systems this equation can be considered as the quantum mechanical analogue of the classical LL equation with damping parameter *λ*. By following transformation of the time *t* → *t*/(1 + *λ*^2^), it is easy to transfer this LL equation to the Landau- Lifshitz-Gilbert (LLG) one in the limit of a large damping. With this replacing the Schrödinger Eq. (1) finally becomes:

2 $$i\hslash (1+{\lambda }^{2})\frac{d}{dt}|\psi (t)\rangle =(\hat{H}-i\lambda (\hat{H}-{\langle H\rangle }_{t}))|\psi (t)\rangle .$$

Note, this equation can be reformulated quantum mechanically to the so-called Liouville-von Neuman equation for the time-dependent density matrix $\hat{\rho }$ operator defined by wave function |*ψ*〉 = |*ψ*(*t*)〉 of multi-spin system in a pure or mixed state^54^. The Eq. (2) can be solved effectively within numerical scheme, for instance, by means of Runge-Kutta method. The optimal value of damping parameter was chosen to be *λ* = 0.05 in this work.

In contrast to the classical description of the spin system, where each classical spin **S** conserves its length and fulfills precession and relaxation only, the quantum mechanical approach deals with the expectation value of the quantum spin 〈**Ŝ**〉 = 〈*ψ*|**Ŝ**|*ψ*〉 and its norm |〈**Ŝ**〉|. This norm is not necessarily to be constant and is also time-dependent, since the wave function |*ψ*〉 = |*ψ*(*t*)〉 is time-dependent. Thereby, the explicit value of the norm describes the strength of entanglement of spins. If the norm is less than its maximal value |〈**Ŝ**〉| < *ℏS*, the spin system exhibits entanglement with a maximal strength for vanishing norm value. More details can be found in ref.^54^. Besides, there is an other quantity for measuring the entanglement strength of spins in multi-spin system, which is called the von Neumann entropy:

3 $$E({\hat{\rho }}_{1})=-\,Tr({\hat{\rho }}_{1}lo{g}_{2}{\hat{\rho }}_{1})=-\,\sum _{{m}_{1}}\,{\lambda }_{{m}_{1}}lo{g}_{2}{\lambda }_{{m}_{1}},$$

here ${\hat{\rho }}_{1}$ is a reduced density matrix of 1-st spin and ${\lambda }_{{m}_{1}}$ is its *m*_1_-th eigenvalue. The reduced density matrix is calculated from the total density matrix taking a trace over the indices of the other spins:

4 $${\hat{\rho }}_{1}=T{r}_{\mathrm{2,3,...}}(\hat{\rho }\mathrm{).}$$

The von Neumann entropy was introduced as the quantum mechanical analogue to the classical Shannon entropy, which was formulated for the computation and information theory^56^. Following this concept, one can employ the mutual information (MI) in order to quantify quantum correlations (or entanglement) between spins:

5 $$M{I}_{i,j}=E({\rho }_{i})+E({\rho }_{j})-E({\rho }_{i,j}),$$

where *E*(*ρ*_*i*_) and *E*(*ρ*_*i*,*j*_) are single- and double-site von Neumann entropies for *i*-th and *j*-th spins, respectively. Note here, that the mutual information has a meaning as the total amount of correlation between two systems, i.e. the total amount of correlations is equal to the quantum mutual information^57,58^. First direct measurements of mutual information in ultracold bosonic atoms in optical lattices have been recently reported^59^.

In this work the time-dependent Schrödinger Eq. (2) was solved for the different quantum spin chains representing various quantum mediators in a bulk material (strontium-copper oxide) and on the surface (copper-nitride surface layer). For this purpose, the basis set of the equation was chosen as eigenbasis of the total momentum operator of the spin system. The quantum spin Hamiltonian of the system was built in Heisenberg-Dirac-Van Vleck form by means of the irreducible tensor operator technique^60,61^:

6 $$\hat{H}=-\,2\sum _{i}\,{J}_{i,i+1}{\hat{{\bf{S}}}}_{i}\cdot {\hat{{\bf{S}}}}_{i+1}+\sum _{i}\,[{D}_{i}{\hat{S}}_{i,z}^{2}+{E}_{i}({\hat{S}}_{i,x}^{2}-{\hat{S}}_{i,y}^{2})]-g{\mu }_{B}\sum _{i}\,{{\bf{B}}}_{i}\cdot {\hat{{\bf{S}}}}_{i},$$

here **Ŝ**_*i*_ = (*Ŝ*_*i,x*_;*Ŝ*_*i,y*_;*Ŝ*_*i*,*z*_) is the spin operator of the *i*-th cite in a quantum spin chain and which is determined within the spin quantum numbers *S* ∈ {1/2; 1; 3/2; 2}. The first sum in QSH (6) describes the anisotropic exchange interactions of the neighboring spins along chain with ferromagnetic (*J*_*i*,*i* + 1_ > 0) or antiferromagnetic (*J*_*i*,*i* + 1_ < 0) couplings, while the second one represents the uniaxial (*D*_*i*_) and transverse (*E*_*i*_) magnetic anisotropies with the *z*-direction, assuming to be the easy axis of magnetization and the third sum is the Zeeman term, which represents the interaction between the spin system and the external magnetic field **B**_*i*_ = (*B*_*i*,*x*_; *B*_*i*,*y*_; *B*_*i*,*z*_). Here, we have perturbed a spin state of an edge (*i* = 1) spin only by applying the time-dependent external magnetic field *B*_1,*z*_(*t*), which was ascribed by step-like function with a reversible magnetization from ${B}_{z}^{0}$ to $-{B}_{z}^{0}$ along the easy magnetization direction of the spin system. Moreover, we have applied also a short Gaussian magnetic pulse ${B}_{\mathrm{1,}x}(t)={B}_{x}^{0}exp\,[-\frac{1}{2}{(\frac{t-{t}_{0}}{{T}_{w}})}^{2}]$ along *x*-direction, in order to bring the spin system out of equilibrium and to accelerate the spin relaxation processes (for more details see ref.^33^). All the calculations were performed using home-made code and repeated in the Mathematica package.
